# Deep Learning for Automated Delineation of Pediatric Cerebral Arteries on Pre-operative Brain Magnetic Resonance Imaging

**DOI:** 10.3389/fsurg.2020.517375

**Published:** 2020-10-26

**Authors:** Jennifer L. Quon, Leo C. Chen, Lily Kim, Gerald A. Grant, Michael S. B. Edwards, Samuel H. Cheshier, Kristen W. Yeom

**Affiliations:** ^1^Department of Neurosurgery, Stanford University, Stanford, CA, United States; ^2^Department of Urology, Stanford University, Stanford, CA, United States; ^3^Stanford School of Medicine, Stanford, CA, United States; ^4^Department of Neurosurgery, University of California, Davis, Davis, CA, United States; ^5^Department of Neurosurgery, University of Utah School of Medicine, Salt Lake City, UT, United States; ^6^Department of Radiology, Stanford University, Stanford, CA, United States

**Keywords:** pediatric brain, surgical planning, pre-operative magnetic resonance imaging, intracranial vessels, deep learning

## Abstract

**Introduction:** Surgical resection of brain tumors is often limited by adjacent critical structures such as blood vessels. Current intraoperative navigations systems are limited; most are based on two-dimensional (2D) guidance systems that require manual segmentation of any regions of interest (ROI; eloquent structures to avoid or tumor to resect). They additionally require time- and labor-intensive processing for any reconstruction steps. We aimed to develop a deep learning model for real-time fully automated segmentation of the intracranial vessels on preoperative non-angiogram imaging sequences.

**Methods:** We identified 48 pediatric patients (10-months to 22-years old) with high resolution (0.5–1 mm axial thickness) isovolumetric, pre-operative T2 magnetic resonance images (MRIs). Twenty-eight patients had anatomically normal brains, and 20 patients had tumors or other lesions near the skull base. Manually segmented intracranial vessels (internal carotid, middle cerebral, anterior cerebral, posterior cerebral, and basilar arteries) served as ground truth labels. Patients were divided into 80/5/15% training/validation/testing sets. A modified *2-D* Unet convolutional neural network (CNN) architecture implemented with 5 layers was trained to maximize the Dice coefficient, a measure of the correct overlap between the predicted vessels and ground truth labels.

**Results:** The model was able to delineate the intracranial vessels in a held-out test set of normal and tumor MRIs with an overall Dice coefficient of 0.75. While manual segmentation took 1–2 h per patient, model prediction took, on average, 8.3 s per patient.

**Conclusions:** We present a deep learning model that can rapidly and automatically identify the intracranial vessels on pre-operative MRIs in patients with normal vascular anatomy and in patients with intracranial lesions. The methodology developed can be translated to other critical brain structures. This study will serve as a foundation for automated high-resolution ROI segmentation for three-dimensional (3D) modeling and integration into an augmented reality navigation platform.

## Introduction

Brain tumors are often devastating, life threatening, and permanently affect quality of life. The mainstay of treatment for many brain tumors is surgical resection, with more extensive resections showing a clear benefit on survival (1). However, surgical resection is especially challenging when tumors distort the intracranial arteries by compressing or growing around them. The extent of resection can be limited by the involvement of the arteries, which, if removed or damaged, can lead to death or devastating disabilities.

Neurosurgeons utilize intraoperative navigation systems when large tumors have severely distorted the intracranial arteries, as these systems are useful adjuncts for executing nuanced surgical plans, as well as confirming the extent of resection. However, standard intraoperative navigations systems are limited: they are based on two-dimensional (2D) representations of three-dimensional (3D) anatomy and provide guidance using flat screen monitor visualization. While some navigation systems have limited 3D reconstruction capabilities, most require manual segmentation of any regions of interest (ROIs, e.g., eloquent structures to avoid or the tumor being resected). Image-by-image identification of ROIs within a large series could include hundreds to thousands of individual images, each with multiple ROIs. This demarcation is time-consuming and labor intensive such that most neurosurgeons usually rely on quick 2D representations rather than forming 3D plans that are anatomically more accurate (2).

Current automated systems for identifying ROIs rely on contrast or time-of-flight sequences and use pixel intensity thresholds, which do not always identify normal anatomic structures or pathologic lesions to the fidelity that is needed for intraoperative neurosurgical navigation (3). Additionally, these algorithms often require manual pre-processing, are computationally intensive, and have not yet translated to real time use in clinical practice. Studies have applied various machine learning approaches to delineate the cerebral vasculature using vascular MRI sequences (3–5), but no study has used routine clinical pre-operative MRIs to delineate neurovasculature. Furthermore, many of these models apply to adult anatomy, and thus may not translate to the pediatric population. For instance, in young children, age-related differences in myelination may affect image intensity and thus make automated methods more challenging than in the adult population.

Deep learning, a form of machine learning that is task-oriented rather than reliant on a priori selected spatial or intensity features, has shown promise in rapid image classification and segmentation tasks (6–8). We present a deep learning U-net model for the automated segmentation of the main intracranial arteries by training on pre-operative structural MRI scans of pediatric patients with anatomically preserved vasculature as well as those with lesions compressing or encompassing the cerebral arteries. This model has the potential to facilitate intraoperative navigation with minimal human oversight through high-throughput, automated labeling of neurovasculature without the need for contrast images or dedicated vascular sequences.

## Materials and Methods

### Patient Selection

3D isovolumetric T2-weighted pre-operative MRIs were retrospectively collected from consecutive patients <25 years old undergoing work-up between 2011 and 2018. These included patients with normal intracranial vascular anatomy undergoing evaluation of extracranial pathology, concussion, or surgical planning for non-lesional epilepsy, as well as those undergoing surgical planning for intracranial lesions. All images were reviewed by a board-certified pediatric neuroradiologist (KWY) as well as a board-certified pediatric neurosurgeon (SHC) and confirmed to have the requisite pre-surgical imaging protocol suitable for integration into a surgical navigation system.

Institutional review board (IRB) approval was obtained for the acquisition and analysis of all clinical and imaging data (*Stanford IRB-44851*). Informed consent was obtained at the time of imaging acquisition but waived by the IRB for the retrospective review since images were de-identified immediately after collection and prior to analysis.

### MRI Acquisition

MRI was performed at 3T using the following magnets: GE Discovery, GE LightSpeed, GE Revolution (GE Healthcare, Waukesha, WI). The 3D isovolumetric isotropic T2-weighted MRI protocol comprised of Freq FOV 24; TR/TE 2500/MAX; ETL 100; slice thickness 0.5–1 mm; matrix (512 × 512); BW 62.50.

### Ground Truth Labels

The main intracranial arteries [internal carotid (ICA), anterior cerebral (ACA), middle cerebral (MCA), posterior cerebral (PCA), basilar, anterior communicating (Acom), posterior communicating (Pcom) arteries] were manually segmented by a neurosurgery resident (JLQ) on axial images from the level of the cavernous sinuses inferiorly to the frontal horns superiorly. Segmentations were confirmed by a board-certified pediatric neuroradiologist (KWY).

### Image Processing and Data Augmentation

DICOM images and their manual segmentations were processed using the Python programming language with the pydicom and SimpleITK packages (9). Images were resampled to 512 × 512 pixels in the axial dimension. Nyul histogram normalization was performed on all of the images using the intensity distributions from the images in the training set (10). Axial slices were randomly flipped, rotated, translated, and cropped to a final size of 480 × 480 pixels for data augmentation, to improve the generalizability of the model. 3D projections were rendered using ITK-SNAP.

### U-Net Model Architecture

**Figure 1:**
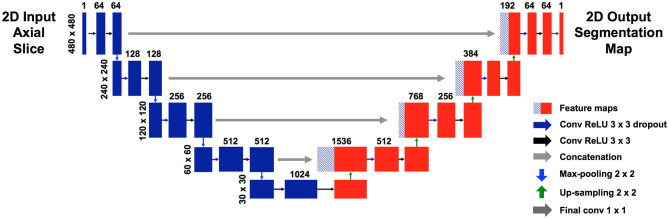
Modified U-net architecture. Axial slices were cropped to 480 × 480 pixels and fed in as input. The model outputs, for each pixel, a probability that the pixel is a blood vessel. Probabilities were thresholded at 0.5.

We used a modified U-net (Figure 1), which is a convolutional neural network (CNN) as described by Ronneberger et al. (11). Our U-net accepts an input of 480 × 480 pixels. Each level of the encoding half (left side) applies two padded 3 × 3 convolutional layers with stride 1, followed by a rectified linear unit (ReLU) and a 2 × 2 max-pooling operation with stride 2. Each layer reduces the dimensions by half, and the number of feature channels is doubled. The final, fifth level includes two 3 × 3 convolutional layers without a pooling layer. The decoding half (right side) recovers the original dimensions of the input images by up-sampling the feature maps and concatenating the corresponding feature channels from the each layer in the encoding half, followed by ReLU. The final layer is a 1 × 1 convolution that decodes the feature vector into a probability prediction for each pixel (vessel vs. non-vessel).

### Data Split and Model Training

MRIs were allocated into development (training and validation) and held-out test sets using random stratified sampling within each group. The breakdown was 80/5% for the training/validation and 15% for the held out test set. The model was trained to minimize the generalized dice loss as described by Sudre et al. (12). The weighted generalized dice loss (Equation 1) was used given the heavy imbalance of vessel to non-vessel pixels. Training was performed via stochastic gradient descent for 100 epochs. A hyperparameter search was performed using the validation set: learning rate = 3e-4, batch size = 2, dropout = 0.2. The final chosen model was the one with the lowest loss on the validation set, to minimize overfitting.

Equation 1: Generalized dice loss (GDL) (12). Subscript 1 subscript indicates vessel. Subscript 0 indicates non-vessel. *l* = label, *p* = predicted. The weight *w* = 1/*n*^2^, where *n* is the number of respective pixels (vessel or non-vessel) for that axial slice.

$$\begin{array}{l} GDL=\;1-\frac{2\left[w_{0}\sum \left(l_{0}{\;\times \;p}_{0}\right)+w_{1}\sum \left(l_{1}\;\times \;p_{1}\right)\right]}{w_{0}\sum \left(l_{0}+p_{0}\right)+w_{1}\sum \left(l_{1}+p_{1}\right)} \end{array}$$

### Model Evaluation

We assessed segmentation accuracy using the Dice coefficient (Equation 2) (13), a measure of the overlap between the blood vessel pixels delineated on the ground truth label compared to the predicted pixels by the model.

Equation 2: Dice coefficient (13). TP = true positive, FP = false positive, FN = false negative.

$$\begin{array}{l} Dice=\frac{2\;\times \;TP}{\left(2\;\times \;TP\right)+FP+FN} \end{array}$$

## Results

### Intracranial Vessel Dataset

Patients undergoing evaluation at Lucile Packard Children's Hospital from 2011 to 2018 were included. A total of 50 patients were found to have high resolution, isovolumetric T2 MRIs. Isovolumetric T2 MRIs were chosen given the ability to reliably identify and visualize anatomical structures including the cerebral vasculature. The following two patients were excluded: one with prior tumor resection (*n* = 1) since vessels may have been coagulated with subsequent angiogenesis; and one with a brainstem vascular lesion (*n* = 1) since they may have had obscured and potentially abnormal vessels. The final dataset included 48 patients aged 10-months to 22-years old (median = 9.5), with 21 girls and 27 boys. These comprised of 28 patients undergoing surgical planning for non-lesional epilepsy (23), evaluation of extracranial pathology (3), or concussion (2). We also included 20 patients with intracranial lesions, some of whom had significant distortion or encompassment of the intracranial vessels (15). Intracranial lesions included craniopharyngioma (4), ganglioglioma (4), pilocytic astrocytoma (3), ependymoma (1), oligodendroglioma (1), subependymal giant cell astrocytoma (1), tectal glioma (2), epidermoid cyst, (1), porencephalic cyst (1), and primitive neuroectodermal tumor (1). One patient had hemimegencephaly but was categorized with the “lesion” group due to distortion of the intracranial vasculature. All patients with intracranial lesions were imaged prior to surgical intervention. Six patients had also underwent vascular imaging (MR angiography, CT angiography, or digital subtraction angiography), but all after their initial MRI. Patient demographics are shown in Table 1.

**Table 1:** Patient dataset.

**Normal vessel patients**	*N* = 28
Age	10.6 months−22.6 years (median = 9.97)
Male	*N* = 16
Female	*N* = 12
Non-lesional epilepsy	*N* = 23
Extracranial pathology	*N* = 3
Concussion	*N* = 2
**Intracranial lesion patients**	
Age	2–22 years (median = 9.3)
Male	11
Female	9
Craniopharyngioma	4
Ganglioglioma	4
Pilocytic astrocytoma	3
Ependymoma	1
Oligodendroglioma	1
Subependymal giant cell astrocytoma	1
Tectal glioma	2
Epidermoid cyst	1
Porencephalic cyst	1
Primitive neuroectodermal tumor	1
Hemimegancephaly	1

### U-Net Segmentation of Intracranial Vessels

**Figure 2:**
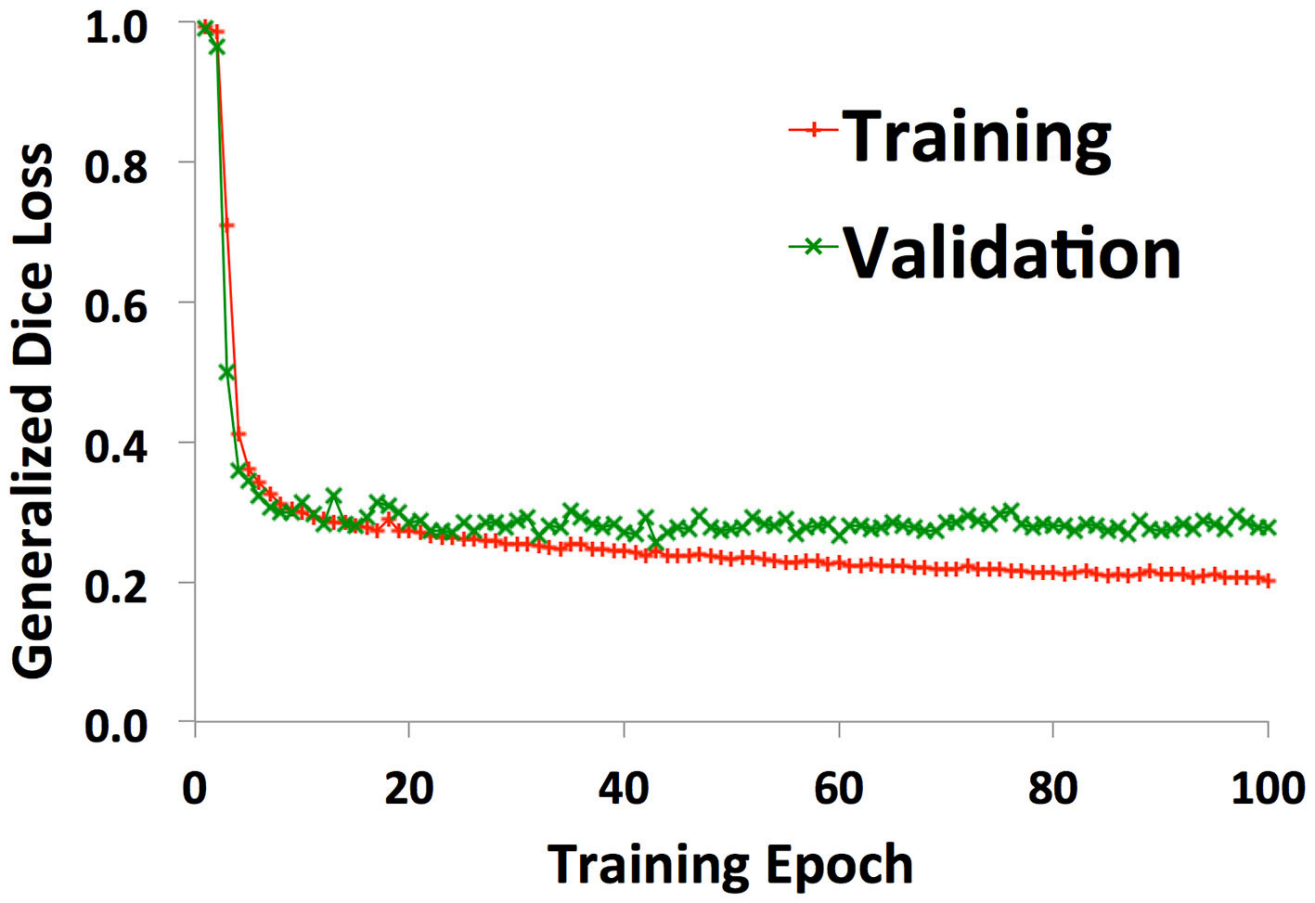
Training curves showing decrease in generalized dice loss for the training (red “+”) and validation (green “x”) as training progressed. The model with the lowest validation loss was chosen for the final model, to reduce overfitting.

38 patients (22 with normal anatomy, 16 with intracranial lesions) were used to train the U-net. A validation set of 2 patients (1 normal, 1 lesion) was used to track training progress and check for overfitting. Figure 2 shows the training curve depicting the decrease in generalized dice loss for the training set as training progressed. Model training took 18 h to complete. The model with the lowest generalized dice loss on the validation set (epoch 43) was chosen as the final model.

**Figure 3:**
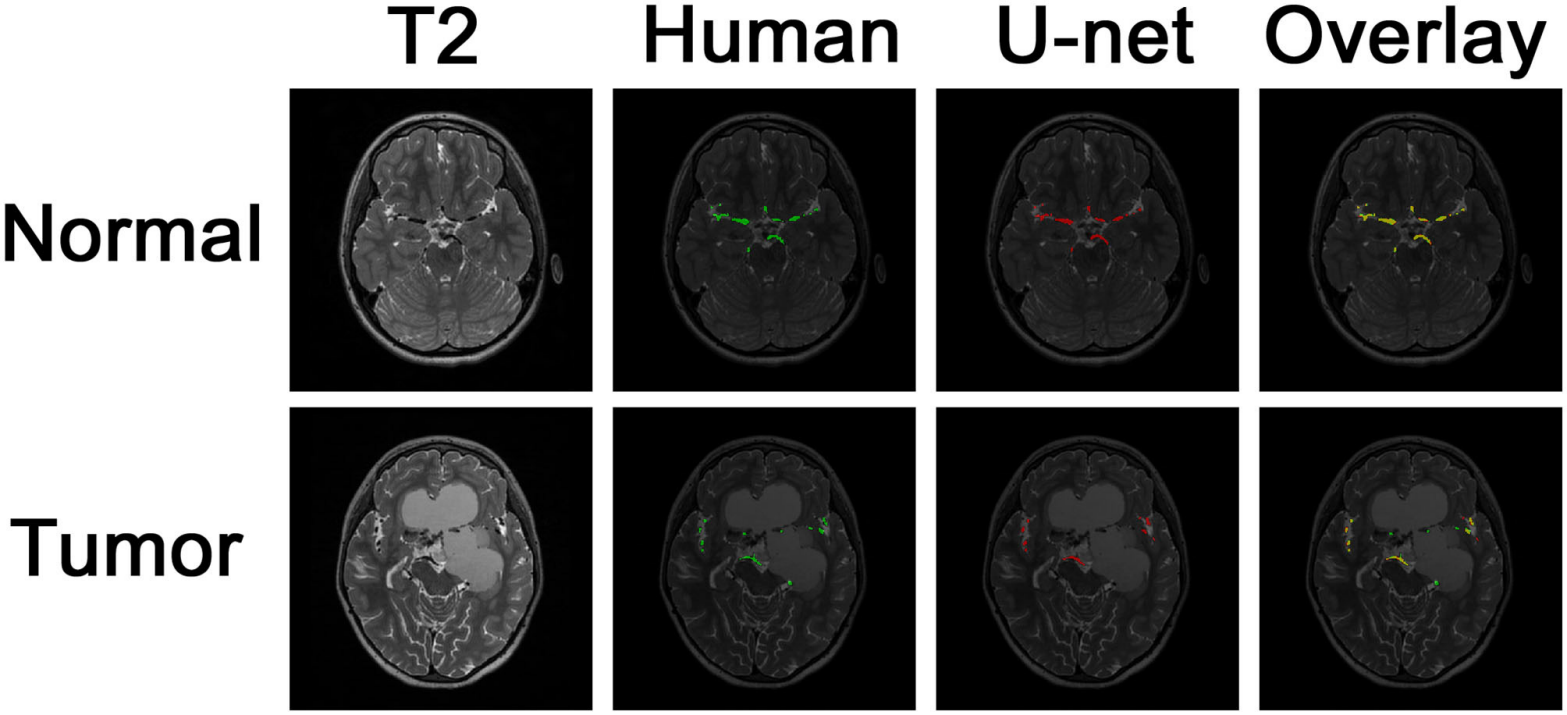
Segmentation of intracranial vasculature by the deep learning model. T2 slices and manual (human) segmentation of vessels are shown for example patients with normal anatomy vs. intracranial tumor. Model (U-net) segmentation is shown in red. An overlay of manual segmentation and model segmentation is shown with green as true positive, yellow as false negative, and red as false positive.

Example images of the original scan, ground truth label, model-generated segmentation, and overlaid figures are shown in Figure 3. Images were generated in under 0.2 s per axial slice, and <8 s per scan. A dice score of 0.75 was achieved on the held-out test set of 8 patients: 5 patients with normal anatomy and 3 patients with intracranial lesions. The normal subset had a dice score of 0.77 and the tumor subset had a dice score of 0.71, indicating better performance of the model on patients with normal vascular anatomy.

### 3D Visualization of the Intracranial Vessels

**Figure 4:**
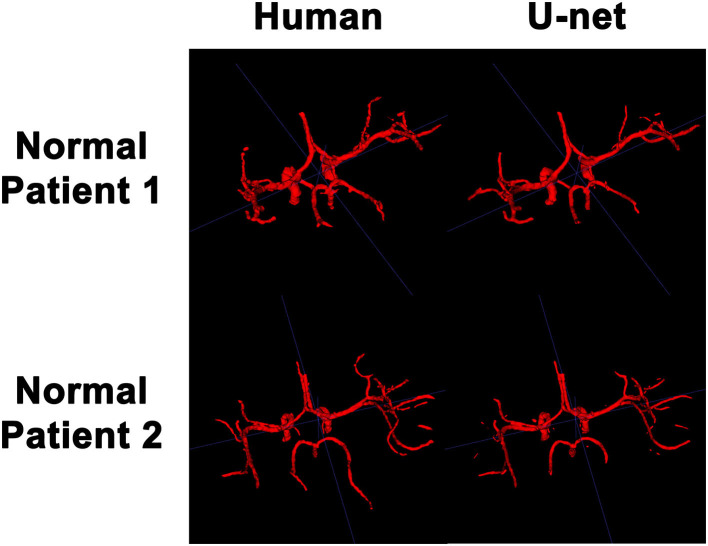
3D reconstruction of intravascular segmentation performed manually by human or automatically by the deep learning model in patients with normal intracranial vasculature.

**Figure 5:**
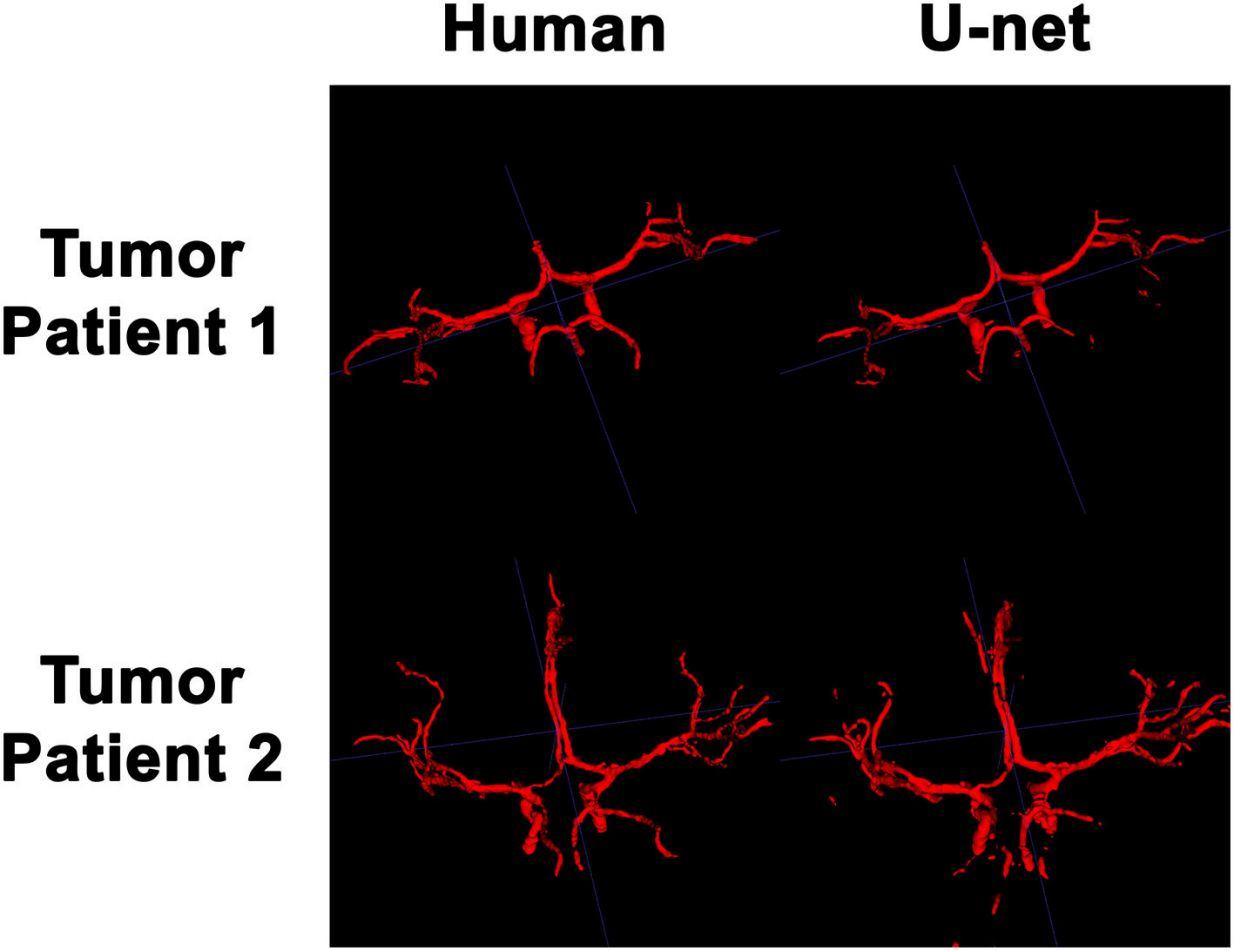
3D reconstruction of intravascular segmentation performed manually by human or automatically by the deep learning model in patients with compressed intracranial vasculature from intracranial tumors.

In order to visualize the intracranial vessels in a manner similar to that of pre-operative planning systems, the axial slices were stacked to generate 3D projections of the vessels in patients with normal vasculature (Figure 4) and with tumors (Figure 5). Qualitatively, the model was able to segment the main arteries of the Circle of Willis with considerable fidelity as reviewed by our neuroradiologist (KWY). The fidelity of the prediction seemed to decrease for the distal branches of the middle cerebral arteries. The tumor scans had more false positive artifacts, as small isolated islands of pixels that do not appear to be vascular structures on the 3D reconstructions.

## Discussion

Our results demonstrate the first application of a CNN to identify and segment the intracranial vasculature using non-contrast, pre-operative brain MRIs from children. The use of deep learning to fully automate intracranial vascular segmentation using standard of care brain MRI scans, without additional contrast or a vascular protocol, carries important clinical implications. These include more efficient segmentation outputs, time and cost savings, as well as the potential for real-time translation into safer surgical resection of tumors in close proximity to critical vascular structures, particularly at the skull base, brainstem, or peri-Sylvian regions of the brain.

While some techniques for automated intra-operative brain volume rendering exist, most require extensive pre-processing steps, including skull-stripping (14). At present, no software specifically targets rendering of the main intracranial vessels, and most only reconstruct the surface vasculature of the brain. Additionally, these techniques require contrast-enhanced scans in order to threshold the signal intensity of brightly enhancing vasculature from background brain (14, 15). With thresholding methods, other high signal regions, such as hemorrhage, protein, or bone marrow, can overlap with and obscure tumor tissue and vessels.

Given that the MRI protocols used in surgical navigation systems are less sensitive to vascular delineation, (16) some investigators have used magnetic resonance angiography (MRA) and intraoperative ultrasound angiography projected as a stereoscopic display overlying the surgical field (17, 18) while others have applied CT angiogram (CTA) with intraarterial contrast injection (16), to better assess vascular malformations. However, such approaches still require surgeons to manually delineate ROIs (tumor or vascular lesions, and anatomic structures of interest) on each frame of the preoperative CT or MRI (19).

Manual delineation and ROI generation by clinical experts remains the gold-standard for intracranial vessel segmentation. While thin-slice 3D MRI scans allow for superior 3D reconstructions for surgical navigation, they have many slices per scan, making manual delineation incredibly time consuming and clinically impractical. Therefore, some surgeons may choose to delineate ROIs on MRIs acquired using more conventional diagnostic protocols. However, due to technical differences in image angle, slice selection, and slice thickness, these ROIs may not accurately transfer onto the 3D reconstructions. Further, any manual delineation would be challenging to perform in real-time, such as during surgery using intra-operative MRIs.

Various studies have examined automated and semi-automated vessel segmentation methods, from rule-based mathematical extraction algorithms to machine-learning techniques (20, 21). One study used intensity thresholds of MRA for computer-aided feature extraction to detect small intracranial aneurysms (22). Mejis et al. applied feature extraction and random forest classification to segment the cerebral vasculature using CTA from stroke patients (23). However, these methods have required separate vascular imaging protocols, such as MRA or CTA (20, 21, 24); and the lengthy pre- and post-processing steps required for these methods pose limitations on real-time clinical implementation.

Deep learning, a task driven form of machine learning, has multiple advantages for clinical implementation. Rather than relying on hand-crafted features, deep learning algorithms *learn* to identify the most relevant features for model optimization. Various studies have shown the utility of CNNs, a form of deep learning, for vessel segmentation, in particular for retinal imaging (20). Another study showed that a single CNN architecture could learn to segment different tissue types using MR brain, MR breast, and cardiac CTA, but did not target the cerebral vasculature (25). Livne et al. demonstrated CNN-based cerebral vascular segmentation using MRAs from 66 adults with cerebrovascular disease. While such results are promising, MRA is often vulnerable to flow-related artifacts (26) and can underestimate the vasculature if there are alterations in blood flow dynamics. Studies have also shown that MRA alone may be inadequate for presurgical neurovascular localization, which has prompted methods for fusing MRA and contrast-enhanced MRI (5, 26).

Given these weaknesses, we trained a U-net CNN to automate vascular segmentation on pre-operative scans, which allow for wider use of our model with more flexible clinical applications. These high-resolution, isotropic, isovolumetric T2-weighted MRI scans are acquired pre-operatively for mapping of brain tumors or seizure foci due to superior diagnostic capability for detecting small lesions as well as the potential for high-resolution reformatting in any plane (27). Unlike prior approaches, ours does not rely on a time-of-flight vascular sequences thus rendering our approach immune to flow-related artifacts. Additionally, the lack of need for intravenous contrast is an additional advantage, due to rising concerns about gadolinium deposition in the brain and its known associated and unknown risks, particularly in children (28, 29). Unlike previous approaches using thresholding techniques, our model was trained to explicitly perform pixel-wise classification and segmentation of the intracranial arteries, and therefore, is specific to identifying neurovasculature rather than any structure that might display signal intensity within a prescribed threshold range. We demonstrate that even without a dedicated neurovascular protocol or intravenous contrast, automated vascular segmentation is feasible using CNNs. Furthermore, we demonstrate good performance on a pediatric dataset, with a wide range of brain sizes from infants to late teenagers, which has not been previously conducted.

With our model, we noticed that segmentation was more accurate for delineating normal vasculature compared to vessels in patients with tumors. This was possibly due to vascular deformation by the tumor. Another possibility is that constriction of the vessels from the underlying mass diminished the overall vascular caliber, which decreases the number of pixels in the denominator of the Dice score on which the model is evaluated, making it quantitatively more sensitive to error. The model also tended to identify more false positives and have more artifacts as the vessels became more distal to the Circle of Willis. One source of false positives may stem from parasitic tumor vessel branches, which were not delineated in our manual ground truth labels that only included the main intracranial arteries. Significantly increasing the size of the training dataset, especially including patients with tumors, would allow the model to better learn which cerebral arteries are consistently represented, thus potentially decreasing the number of false positives.

One major limitation in our study was the small sample size of our retrospective cohort. Despite our rather limited training set of 38 patients, our model was still able to generate relatively high fidelity segmentations of the major intracranial arteries on a held out test set of 8 patients, which were never seen by the model during the training phase. It is likely that more training data would lead to higher accuracy by the convolutional neural network. Since vascular imaging was not available for most of the patients in our study, we did not label our T2 scans using vascular imaging. However, our use of high-resolution, isotropic, isovolumetric scans permitted the best possible visualization of the vascular anatomy, which was labeled by a neurosurgery resident with oversight by a board-certified neuroradiologist, as manual delineation currently remains the gold standard for ROI identification in clinical practice. Future work could include more human labelers to address and to quantify the degree of inter-observer variability in identifying critical structures, as well as the integration of intraoperative MRI sequences to further facilitate the integration of this model into real-time neuronavigation.

## Conclusions

We demonstrate the feasibility and fidelity of a CNN model for segmentation of pediatric cerebral vasculature using a standard of care pre-operative MRI protocol that does not require contrast dye or additional vascular imaging sequences. In future work we hope to incorporate this model into neuronavigational systems for pre-operative planning and intraoperative navigation.

## Data Availability Statement

The datasets generated for this study are available on request to the corresponding author.

## Ethics Statement

The studies involving human participants were reviewed and approved the Stanford Institutional Review Board. Informed consent was obtained at the time of imaging acquisition but waived by the IRB for the retrospective review since images were de-identified immediately after collection and prior to analysis.

## Author Contributions

JQ, LC, LK, SC, and KY: concept and design. JQ and LK: acquisition of data. LC: model design and development. JQ and LC: data analysis and data interpretation. JQ, LC, LK, GG, ME, SC, and KY: manuscript drafting and approval. All authors contributed to the article and approved the submitted version.

## Conflict of Interest

The authors declare that the research was conducted in the absence of any commercial or financial relationships that could be construed as a potential conflict of interest.
